# *Game Changers*: A participatory action research project for/with students with disabilities in school sport settings

**DOI:** 10.3389/fspor.2023.1150130

**Published:** 2023-04-06

**Authors:** Daniel B. Robinson, Sebastian Harenberg, William Walters, Joe Barrett, Anna Cudmore, Kelsey Fahie, Tricia Zakaria

**Affiliations:** ^1^ Department of Teacher Education, St. Francis Xavier University, Antigonish, NS, Canada; ^2^ Department of Human Kinetics, St. Francis Xavier University, Antigonish, NS, Canada; ^3^ Department of Educational Studies, Brock University, St. Catherines, ON, Canada; ^4^ Physical and Health Education Canada, Ottawa, ON, Canada

**Keywords:** sport, disability, adapted sport, school, participatory action research

## Abstract

**Introduction:**

Although school communities may be required to provide physical education opportunities for all students—including for those with disabilities—the same is not generally true with respect to *school sport* (i.e., participation in interscholastic or intramural sport programs). Hence, opportunities for inclusive school sport participation are consequently limited. Recognizing the need for continued attention and action in this area, we recently developed and piloted *Game Changers*—a participatory action research project. Together, 27 students with various cognitive and/or intellectual disabilities (i.e., student-participants), their schools’ six physical education teachers and learning support teachers (i.e., champion-participants), four university researchers (i.e., researcher-participants), and two community partners [i.e., Physical and Health Education (PHE) Canada, Special Olympics Nova Scotia] engaged in the *Game Changers* project with three idealized goals: (a) to bring to the fore para/adapted/inclusive sport opportunities for all students; (b) to provide an empowering opportunity for students with disabilities to participate, make choices, and act as leaders in the development of sport programming; and (c) to engage youth with disabilities in sport as participants, leaders, mentors, and role models.

**Methods:**

Utilizing a mixed-methods design, data were collected from a variety of sources before the implementation of the *Game Changers* program, during its implementation, and once it was complete. These four data sources included the following: pre- and post-program survey for student-participants, pre- and post-program focus group interviews for student-participants, pre- and post-program focus group interviews for champion-participants, and school/sport observations.

**Results:**

The first cycle of this participatory action research project has yielded positive and informative findings. Strictly positive findings, among others, relate to the following: improving upon students' perceived competence and autonomy, inviting student voice, identifying and responding to sport participation barriers, and creating genuine sport opportunities within school settings. More undesirable yet informative findings, among others, relate to the following: unachieved intrinsic motivation and belonging, (un)sustainability of sport programs without “interventions” like *Game Changers*, recreation/leisure as “substitutes” for sport, and a continued want for authentic leadership and mentorship opportunities.

**Discussion:**

With these findings, we offer insights for future iterations of *Game Changers* (and programs like it) in similar school communities.

## 1. Introduction

That youth with disabilities have less favorable and fewer sport experiences than do youth without disabilities is beyond dispute. Certainly, youth with disabilities face a host of barriers not faced by their peers without disabilities (1, 2). These barriers include, for example, a lack of awareness by others about how to include those with disabilities adequately or meaningfully, limited opportunities and programs for training and competition, and difficulties identifying, locating, and utilizing suitable material and human resources (3–5).

While school communities are required to provide *physical education* opportunities for all students—including for those with disabilities—the same is not generally true with respect to *school sport* (i.e., participation in interscholastic or intramural sport programs). Hence, opportunities for inclusive school sport participation are consequently limited. These limited opportunities are impacted by additional barriers somewhat unique to school contexts. For example, with respect to interscholastic sport programs, teacher-coaches may be inadequately trained to address the needs of students with disabilities, students with disabilities may only be afforded “exhibition” competition opportunities at sport events, and school sport teams are oftentimes prohibitively traditional and/or highly selective to the best performing student-athletes (6, 7). Though intramural sport programs do not face some of these same barriers to the same degree, they do face some barriers, nonetheless. For example, youth with disabilities have less favorable and fewer opportunities to participate in intramural sport programs due to normalized traditional intramural sport activities, concerns about acceptance from peers, and a hidden need for labor on the part of parents/guardians (8–10).

While youth with disabilities face these sorts of barriers to sport participation, there is documentary evidence suggesting that such sport participation has the potential to provide many benefits. For example, sport participation can improve academic success, build self-esteem, prevent various health issues, and improve upon general quality of life (11–13). Additionally, sport participation can help youth with disabilities develop skills related to teamwork, goal-setting, and other goal-oriented behaviors (6). The benefits to be gleaned by such sport participation are not limited to those with disabilities. For example, “integrated” sport programs can positively impact participants' coaches and relatives as well as their participating peers without disabilities (14). Welcoming students without disabilities into para/adapted/inclusive activities also has a positive impact upon their own perceptions of “others” with disabilities and with disability generally (15).

Not only would youth with disabilities benefit from sport participation within their school communities, but they also ought to be entitled to it. That is, while in Nova Scotia all students are meant to enjoy the basic right to “full and equal participation in education” (16, para 1), the same is not yet also true with respect to extracurricular school sport (i.e., participation in interscholastic or intramural sport programs). Until such sport participation is engrained as a right for all students, it will continue to fall upon champions to actualize what is not yet mandated.

Given this milieu, pioneering initiatives and research projects aimed at improving opportunities and building knowledge related to sport and youth with disabilities are observably important endeavors. Moreover, given both the range of possible contextual factors (e.g., nature of various disabilities, sport experience possibilities) as well as the importance of responding to the needs of the specific individuals within these various contexts (i.e., Nihil de nobis, sine nobis), “one-size-fits-all” approaches must be eschewed in favor of more context-dependent and context-responsive ones.

Recognizing the need for continued attention and action in this area, we recently developed and piloted *Game Changers*—a participatory action research project [see (17)]. Together, students with various cognitive and/or intellectual disabilities (i.e., student-participants), their schools' physical education teachers and learning support teachers (i.e., champion-participants), university researchers (i.e., researcher-participants), and two community partners [i.e., Physical and Health Education (PHE) Canada, Special Olympics Nova Scotia] engaged in the *Game Changers* project with three idealized goals: (a) to bring to the fore para/adapted/inclusive sport opportunities for all students; (b) to provide an empowering opportunity for students with disabilities to participate, make choices, and act as leaders in the development of sport programming; and (c) to engage youth with disabilities in sport as participants, leaders, mentors, and role models.

## 2. Literature summary

### 2.1. Youth with disabilities and barriers to sport participation

Crawford and Godbey (18) provided three categories of constraints that impede or constrain opportunities for leisure generally—those that are intrapersonal, interpersonal, and structural. This framework has been applied to explore barriers to sport participation for those with disabilities, in both general and specific contexts [e.g., see (19, 20)]. Intrapersonal barriers include such things as low levels of self-efficacy and negative self-attitudes about participating in sport, among others (21–23). Interpersonal barriers include such things as a lack of support from others (including from peers), as well as perceived negative societal attitudes (24–26). Examples of structural barriers include limited accessible information, inadequate facilities, a lack of trained leaders, and prohibitive costs (4, 24, 26–29). Given these three constraint categories' recognizable alignment with social ecological frameworks or socio-ecological models within the physical activity and wellness literature [e.g., see (30–32)], categories of consideration might be further conceptualized in this manner: intrapersonal, interpersonal, and structural (institutional, community, policy). Such attention to a social ecological framework or socio-ecological model ought to embrace this underlying premise: these levels are “interdependent and can exert direct effects on one another” (29).

### 2.2. Youth with disabilities and benefits of sport participation

Just as barriers to sport participation can be framed by qualifying interpersonal, intrapersonal, and structural categories or levels (18), so too can some of the benefits derived from it—particularly as they relate to individuals. For example, intrapersonal benefits of sport participation include improved physical and mental health, self-efficacy, and feelings of athletic competence (33, 34). Interpersonal benefits of sport participation include important social outcomes, such as stronger interpersonal relationships, authentic opportunities for belonging, and genuine companionship (12, 35, 36). Many of these intrapersonal and interpersonal benefits are directly related to health-related *quality of life* indicators; consider, for example, how improved self-perceptions and self-worth, alongside greater opportunities for social acceptance and inclusion, might enrichen the lives of students with disabilities within and outside of sport (33). Additionally, research has found that youth with disabilities who participate in sport enjoy largely sport-specific benefits that are difficult to find elsewhere, including experiencing the “thrill of competition,” bonding with teammates, and identifying and celebrating success by, for example, “winning medals” and “being rewarded with certificates, ribbons and trophies” (35). These and other benefits suggest that offering sport opportunities to youth with disabilities is a necessary undertaking.

### 2.3. Para/adapted/inclusive sport

Given these barriers and benefits related to sport participation, parasport, adapted sport, and inclusive sport programs have been developed and introduced to meet the sport needs and interests of those with various disabilities. These similar and overlapping sporting contexts are unique in that they are meant to be purposefully responsive to the needs and interests of those who have been historically marginalized based on (dis)ability. Parasport, also known as disability sport, is used to describe sport for people with various physical, visual, and/or intellectual impairments (37). Parasport is also used as a synonym, by some, for Paralympic sport (38). There exists a lack of research and understanding about parasport, largely due to several underlying challenges [e.g., variation of integration within mainstream sport, a lack of disability-specific knowledge, limited coaching expertise and pathways; (38)]. Adapted sport refers to sport that is modified or created to meet the unique needs of individual athletes [wheelchair basketball and goalball are both adapted sports; (39)]. Adapted sport includes parasport/disability sport—which typically focuses upon segregated participation (39). Adapted sport focuses on sport modification rather than on the disability, it encourages participation in the most “normal” and integrated environment, and it provides opportunities for the pursuit of excellence in sport through a spectrum of possible settings (39). Adapted sport, too, suffers from a paucity of research, particularly as it relates to motivations, facilitators, and barriers to sport participation (40). These overlapping constructs—parasport and adapted sport—both fall under the broader category of inclusive sport (20).

## 3. Methodology and methods (participatory action research)

**Figure 1:**
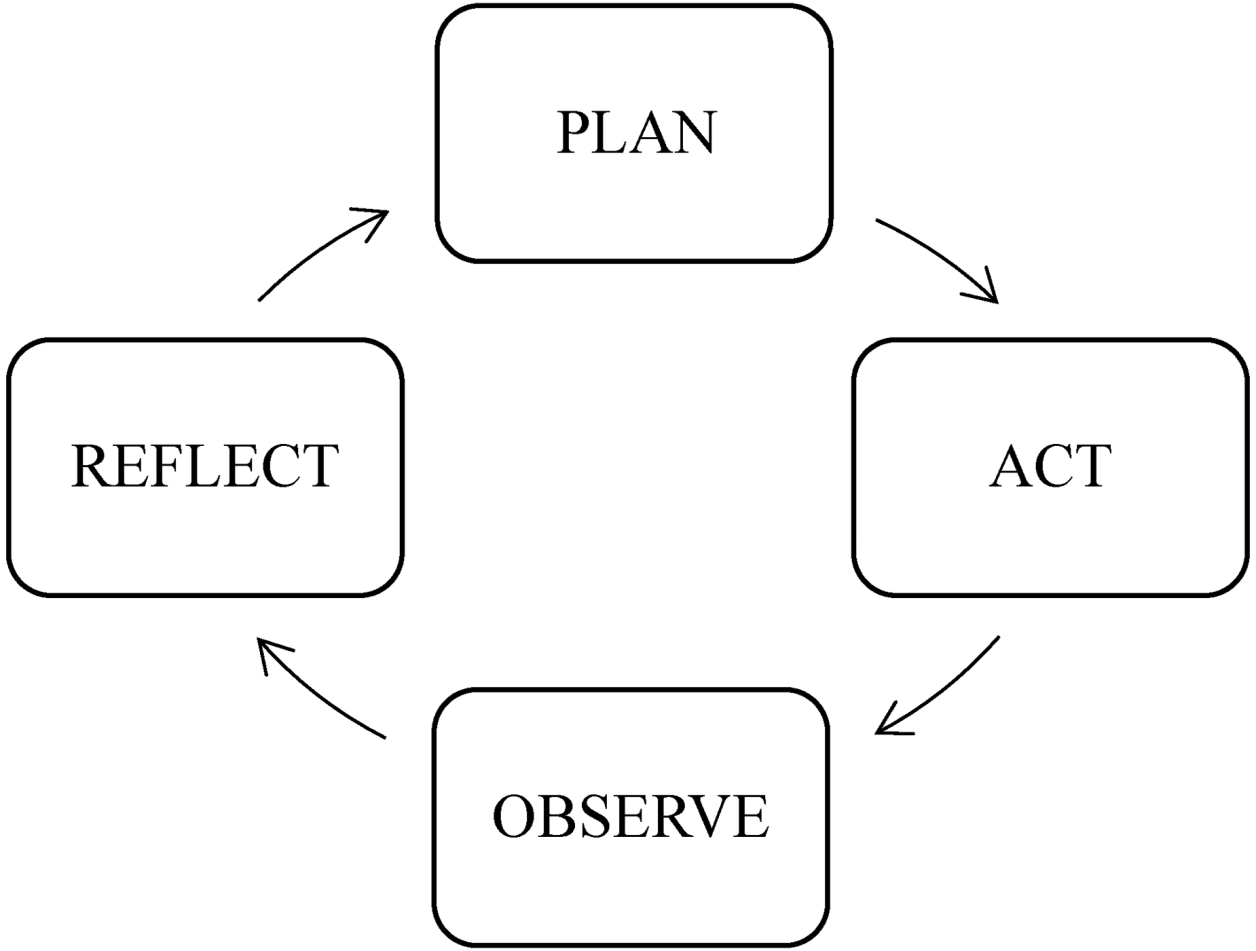
Action research's process.

Action research “often starts small, is participatory, collaborative and inclusive” (41). It is these features that enable attention to small unique contexts, invite insiders and their perspectives, and provide the conditions for creating meaningful communities of change agents. Lewin's (42) initial introduction of action research offered a cyclical process of “actioned” research with five sequential and recurrent stages: think, plan, act, evaluate, and reflect. This process, first introduced over a half-century ago, features in all applications of action research since, though sometimes as the four (similar) stages of planning, acting, developing, and reflecting—or planning, acting, observing, and reflecting (see Figure 1). These four “moments” are dynamic and interconnected (43). It is this process that enables constant and immediate responsiveness to information gleaned from observation and reflection. Lastly, and as offered by Kemmis and McTaggart (44),

action research is a form of collective self-reflective enquiry undertaken by participants in social situations in order to improve the rationality and justice of their own social or educational practices, as well as their understanding of these practices and the situations in which these practices are carried out. (p. 1)

So, action research sets out to change people's practices and the situations in which they do those practices, as well as our understandings of those practices (45).

Participatory action research is a type of action research that is people-centered and power-conscious (43). It is somewhat unique (from other types of action research) in that it, by design, offers “participants” opportunities to share and create conditions for themselves to actively develop forms of action that are in response to their own unmet needs or undesirable conditions, while also co-building and establishing communities of practice (17).

### 3.1. Procedures

The *Game Changers* participatory action research's first cycle had six stages, occurring over a period of just under one year (see Table 1). (This period was limited by multiple COVID-19-related interruptions and cancellations.) Initially, the researcher-participants partnered with the country's recognized national leader in physical and health education (PHE Canada) as well as with a provincial organization committed to enriching the lives of those with disabilities through sport (Special Olympics Nova Scotia). Together, PHE Canada and Special Olympics Nova Scotia completed a participant and environmental scan before developing a framework for the *Game Changers* program. This framework development informed the construction of a one-day participant workshop (for student-participants and champion-participants) built upon principles of human-centered design (HCD)—an increasingly adopted method for inviting stakeholders to co-create innovative and responsive solutions to their own challenges (46).

**Table 1:** Overview of stages, dates, participatory action research (PAR) steps, specific activities, and participants and partners.

Stages, Dates, PAR Steps	Specific Activities	Participants, Partners
**Stage 1 Dates**: Sep.–Dec. **PAR Steps**: reflect, plan	Participant/environmental scan, workshop/resource development	**Participants**: four researchers **Partners**: PHE Canada, Special Olympics Nova Scotia
**Stage 2 Dates**: Jan.–Feb. **PAR Steps**: observe, reflect, plan	Pre-sport program implementation data collection and program planning	**Participants**: 27 students, six champions, four researchers
**Stage 3 Dates**: Feb. **PAR Steps**: reflect, plan, act	Workshop/HCD exercise (*Game Changers* planning)	**Participants**: 27 students, six champions, four researchers **Partners**: PHE Canada, Special Olympics Nova Scotia
**Stage 4 Dates**: Feb.–Jun. **PAR Steps**: act, observe, reflect	Sport program implementation (and observations)	**Participants**: 27 students, six champions, four researchers
**Stage 5 Dates**: May–Jun. **PAR Steps**: observe, reflect, plan	Post-sport program implementation data collection	**Participants**: 27 students, six champions, four researchers
**Stage 6 Dates**: Jun.–Jul. **PAR Steps**: reflect	Knowledge mobilization	**Participants**: four researchers **Partners**: PHE Canada, Special Olympics Nova Scotia
**Stage 7 Dates**: Jul.–Aug. **PAR Steps**: reflect, plan	Plan *revised* pre- and post-sport program implementation data collection and workshop/HCD exercise	**Participants**: four researchers **Partners**: PHE Canada

This one-day workshop was hosted at each of two participating secondary schools. Student-, champion-, and researcher-participants were all in attendance. The goal of the workshop was to provide students with the knowledge and tools needed to create a school-based sport program that suited their needs and aligned with their interests. The first half of the workshop was dedicated to knowledge sharing and learning about topics such as inclusion, leadership, physical literacy, and adapted physical activity. The second half of the workshop focused on the ideation and creation processes of HCD where students worked together to brainstorm programming options that motivated them to participate. Importantly, student-participants and champion-participants also identified (intrapersonal, interpersonal, and structural) barriers to school sport participation. Together, and with financial support provided by Sport Canada, student-participants and champion-participants considered these idealized programming options and possibilities for overcoming existing barriers so that they could implement “tailor-made” sport programs in their school communities.

These tailor-made sport programs included several different sport-related activities. To address some of the student- and champion-identified intrapersonal and interpersonal barriers (e.g., related to perceived limited/low abilities, negative sport attitudes, lack of support from others), “coaches” were recruited [educational assistants (EAs) and student leaders], students were afforded significant license regarding what “counted” as “sport” activities, and students were able to opt in or out, as they wished, of activity participation. To address some of the champion-identified structural barriers (e.g., related to limited expertise, resources, and facilities), experts were hired for some activities, material resources and uniforms were purchased for students, and inside-of-school and outside-of-school facilities were booked as needed. With this attention to these barriers and through the collaborative efforts of all participants, students were able to go from nearly no school sport to a full sport experience. These were largely adapted sport offerings (e.g., swimming, volleyball), though some were done alongside peers without disabilities in an inclusive (integrated) sport context (e.g., track and field, Unified Sports).

Before participating in this one-day participant workshop and HCD exercise, data were collected from student-participants and champion-participants (detailed below) before the full implementation of the *Game Changers* program. They then did the same after *Game Changers* sport opportunities were complete. Lastly, the final stage included knowledge mobilization, dissemination, and refinement efforts. These final “reflection” efforts informed a subsequent cycle with additional participants in a new location (see Stage 7 in Table 1, below).

The specific action research activities undertaken by the various participants and partners highlight our efforts to share or distribute power throughout the entire *Game Changers* exercise. Certainly, student-, champion-, and researcher-participants all played important roles in all four of the action research moments (i.e., plan, act, observe, reflect). These power sharing efforts were never really meant to “equalize” that power distribution. What was intended was for students and champions to take leading roles in designing sport programs, and for their own experiences and observations to be prioritized (through data collection measures).

Notwithstanding our efforts to prioritize students' (and champions') experiences and observations through data collection measures, we do appreciate that these participants' voices were not principal ones with respect to deciding upon and carrying out these data collection measures. This, in some ways, presents a shortcoming of our action research process—one that required an articulation (and Research Ethics Board approval) of data collection measures *before* we met with students and champions.

Despite our best efforts, power sharing was nonetheless oftentimes both unpredictable and necessarily flexible; these happenings were certainly consistent with what is typical within participatory action research (47). Indeed, at times, efforts towards power sharing amongst participants were occurring while we were—what Herr and Anderson (48) have aptly described as—“designing the plane while flying it” (p. 69).

### 3.2. Participants

Understanding that there are limited-to-no opportunities for students with disabilities to participate in school sport within many school communities (6–10), partners PHE Canada initially solicited expressions of interest from potential school champions from their own network of “connected” teachers (i.e., those who had some sort of established relationship with PHE Canada; e.g., PHE Canada member, attended a PHE Canada conference). The then-potential school champions (and their school communities) had to meet three criteria: they had to commit to participating in *Game Changers* as a champion-participant, their school community had to have an observable “deficit” with respect to sport participation opportunities for students with disabilities, and their school community had to welcome a student-informed process to attend to this deficit so that their students with disabilities might engage in newly co-created and context-dependent sport opportunities.

The four researcher-participants were from two different universities, in two different Canadian provinces. Champion-participants (and student-participants) were from two different senior high schools in one large Canadian city. Champion-participants from Jones Academy included one physical education teacher, one learning support teacher, and one learning support pre-service teacher while champion-participants from Kent Academy included two physical education teachers and one learning support teacher. At both sites, EAs and student leaders were available, and helpful, during several of the participatory action research project's specific activities (e.g., workshop and HCD exercise, sport program implementation).

The 27 student-participants included 13 students from Jones Academy who were in grades 9–12. They had various cognitive and/or intellectual disabilities and most had low support needs. The additional 14 students from Kent Academy were in grades 10–12. They also had various cognitive and/or intellectual disabilities and some had low support needs, and some had high support needs. *Game Changers* was initially conceptualized as a sort of sport participation “intervention” that would “target” *all* students with disabilities within participating school communities. So, although all students with disabilities were invited to participate in *Game Changers* (e.g., including those with physical disabilities), only those with various cognitive and/or intellectual disabilities actually participated in the program (and note, the gross majority had cognitive disabilities). So, it is important to recognize that though participants are identified herein as having cognitive and/or intellectual disabilities, this identification is, by no means, meant to suggest this was a homogenous group of students (i.e., student-participants' disabilities included autism spectrum disorder and Down syndrome, among others).

### 3.3. Data sources (collection and analysis)

Data were collected from a variety of sources, before the implementation of the *Game Changers* program, during its implementation, and once it was complete. These four data sources included the following: pre- and post-program survey for student-participants, pre- and post-program focus group interviews for student-participants, pre- and post-program focus group interviews for champion-participants, and school/sport observations.

#### 3.3.1. Pre- and post-program survey for student-participants

Before and after the implementation of the *Game Changers* program, student-participants completed the same 48-item survey. The pre-survey was completed one to two weeks before the *Game Changers* program began, and the post-survey was completed one to two weeks after the *Game Changers* program ended. The survey included five subscales: autonomy, belonging, intrinsic motivation, perceived competence, and participation. The subscales of the survey were selected from valid and reliable measures used previously by researchers [see (49–53)]. Student-participants completed these surveys with their schools' champion-participants or with their parents/guardians (i.e., one-on-one). All subscales showed sufficient internal consistency (Cronbach's *α* > .75). Data from these surveys were first analyzed for normality before the use of parametric statistics were considered. As all variables were sufficiently normally distributed, the aggregated scores were compared from pre- to post-program using paired samples t-tests with Cohen's *d* effect sizes. An alpha level of .05 was set for the quantitative analyses.

#### 3.3.2. Pre- and post-program focus group interviews for student-participants and champion-participants

Before and after the implementation of the *Game Changers* program, focus group interviews were also completed with student-participant groups and champion-participant groups. These focus group interviews focused upon barriers and facilitators for sport participation, as well as students' intentions, motivations, and participation in sport events. These focus group sessions also invited input about the *Game Changers* program. Examples of prompting questions included the following:

• In what ways do you and/or other teachers and/or coaches support students/athletes with various disabilities so that they can participate in school sports? (champion-participant question)
• Do you think school sports facilities enable all people—those with and without disabilities—to participate fully and equally? Explain. (champion-participant question)
• Are there any other activities you would like to try participating in, in school sports? What needs to happen for you to do this? (student-participant question)
• How has your participation in *Game Changers* impacted your participation in school sports? (student-participant question)

Champion-participant focus groups were completed online *via* Zoom or in-person and ranged from 55 to 110 min in length. Student-participant focus groups were completed in-person and ranged from 40 to 65 min in length. EAs were present for some of these student-participant focus groups, as necessary. These EAs were helpful at making student-participants feel “at-ease” and at soliciting responses from those who were otherwise shy or quiet. These focus group interviews were audio-recorded and transcribed verbatim. Data from these transcripts were analyzed through an inductive process whereby the initial data were narrowed down to important groups from which participants' perspectives and experiences could be derived (54). The three steps as described by Mills and Gay (54) were completed and repeated during the transcribing process: (a) establishing familiarity with the data and identifying possible themes; (b) describing the data in depth; and (c) categorizing and coding pieces of the data. By coding and categorizing the data according to methods outlined by Creswell (55) and Miles et al. (56), dominant themes emerged, allowing for analysis and interpretation. Searching for commonalities, original insights, and patterns, responses were read multiple times while elements were coded into emerging themes.

#### 3.3.3. School/sport observations

During multiple occasions throughout the implementation of the *Game Changers* program, researcher-participants conducted field observations, whereby they recorded, managed, and subsequently analyzed descriptive and reflective field notes. These observations occurred at the two workshops and at four subsequent sport sessions. Situated as passive observers (57), the researchers recorded descriptive field notes that were factual and comprehensive (e.g., about the setting, participants, dialogues, activities, behaviors). They also included reflective notes related to their thoughts, ideas, questions, and inferences (58). All researchers' field notes were transcribed into observation protocols (59) and, again, these observational data were analyzed by searching for similarities, differences, recurring ideas, clustering, patterns, and relationships in the responses and by coding and categorizing the data [see (55, 56)]. Dominant themes emerged, allowing for analysis and interpretation.

## 4. Results

### 4.1. Survey results

In all, 13 students (response rate 48.1%) completed both the pre- and post-*Game Changers* survey. Ten of those students attended Kent Academy, while three attended Jones Academy. The students were on average 17.53 years (SD = 1.81 years) old. Seven (53.8%) of the students attended grade 12, while three students (23.1%) attended grade 10 and grade 11. The descriptive information of the pre- and post-*Game Changers* survey can be found in Table 2.

**Table 2:** Means and standard deviations of the pre- and post-*Game Changers* survey.

Scale/Question	*α*	Pre-mean (SD)	Post-mean (SD)
Perceived competence	.81	3.96 (1.29)	5.01 (.78)
Intrinsic motivation	.81	5.17 (1.68)	5.25 (1.68)
Autonomy	.82	4.59 (1.51)	5.42 (.91)
Belonging	.78	4.90 (1.26)	5.22 (.89)
Activity school sport		2.18 (1.25)	2.62 (1.45)
Activity lunch time		2.22 (.97)	3.23 (1.30)
Activity evenings		2.58 (1.31)	3.39 (1.26)
Activity weekends		2.10 (.88)	3.46 (1.27)

The survey responses generally indicated favorably significant changes from pre- to post-*Game Changers* assessments. The largest effect size was observable in the domain *perceived competence*, with a mean difference of 1.05 [*t*(_12_)_ _= 3.33, *p* = .006, Cohen's *d* = .92]. Based on the effect size, this finding indicated that the perceived competence increased strongly after the completion of the program. This aligns with te Velde et al.'s (33) research with students with a physical disability; they also found that those who participated in a sport program reported stronger feelings of athletic competence. While only approaching significance, a meaningful increase of the domain *autonomy* was also found [mean difference = .83, *t*_(12) _= 1.97, *p* = .073, Cohen's *d* = .55]. The effect size indicated a medium increase in autonomy. The domains of *intrinsic motivation* and *belonging* did not show significant changes from pre- to post-program evaluation. These results related to *belonging* were surprising, as previous research has shown that interpersonal benefits of sport participation include important social outcomes such as stronger interpersonal relationships, authentic opportunities for belonging, and genuine companionship (12, 20, 35).

For participation, the strongest change was observed in the participation in physical activity on weekends [mean difference = 1.60, *t*_(9) _= 3.36, *p* = .008, Cohen's *d* = 1.06]. The finding indicated that most participants engaged in weekend physical activity once before the program, while they engaged two or three times after the completion of the program. In addition, lunch time activity increased significantly [mean difference = 1.22, *t*(_9_)_ _= 2.82, *p* = .023, Cohen's *d* = .94]. While on average the participants rated their lunch time activity (besides eating) as standing or walking around, they rated it at running or playing a little bit after the completion of the program. While it is not possible to draw a causal relationship here, we recognize that attending to microlevel intrapersonal elements (e.g., skill, perceived competence) has the potential to positively impact physical activity behaviours (30, 31); this may be the case. Other participation questions did not yield significant changes.

### 4.2. Focus group interview results

#### 4.2.1. Focus groups with student-participants

Two focus groups of students from Jones Academy (a total of 13 students) participated in the pre- and post-*Game Changers* focus group interviews. Two focus groups of students from Kent Academy (a total of 12 students) participated in the pre-*Game Changers* focus group interviews and one focus group (with 5 students) participated in the post-*Game Changers* focus group interview. The pre-*Game Changers* interviews highlighted two salient themes: limited sport experiences and perceived barriers to sport participation. The post-*Game Changers* interviews highlighted two salient themes: (mostly) wishing for more and enjoying sport participation.

##### 4.2.1.1. Limited sport experiences

A small number of students at Jones and Kent Academies shared that they had some sport experiences (“I play basketball” and “I play badminton occasionally”). However, many shared that they had limited-to-no sport experiences (“I haven”t really done many sports” and “I have never been on a sports team”) or they provided examples of “non-sport” physical activity engagements as examples of their sport experiences [“I do Just Dance” and “(I like) walks outside”]. Despite this, they were keen to provide examples of some sport experiences they would like to try [“I want to participate in curling,” “(I want) to try swimming,” and “basketball for me over here”]. These findings were not surprising and reaffirmed what we thought to be the case. That is, the finding that there were limited opportunities for sport participation agrees with the existing related research literature (1, 2)—and, indeed, was our initial assumption and impetus for this action research project.

##### 4.2.1.2. Perceived barriers to sport participation

The students were also able to share some of the intrapersonal barriers they believed they faced that hindered their ability to engage in sport, including long-term physical ailments (“I can’t do contact sports”), lack of sport experience [“I don’t have the experience (playing volleyball)”], and limited sport-specific skills (“pitching badly in baseball”). These students recognized intrapersonal barriers that built upon others' similar findings [e.g., (21–23)], though these did provide a more specific and necessary contextualized focus (i.e., addressing a perceived lack of skills).

##### 4.2.1.3. (Mostly) wishing for more

After the *Game Changers* program, students were asked about their subsequent desire to participate in school sport. Some shared that they wished to now participate in more school sport (“I want to do more,” “I want to do more bowling,” and “more swimming”). However, a small number of students did not feel the same way (“I don’t want to do school sports anymore”). Again, McLeroy et al. (30) and Sallis et al. (31), among others, have shown that multi-level attention to socio-ecological factors has the potential to improve physical activity behaviours and physical activity behaviour *intentions*. This is, at least, what we are hopeful for.

##### 4.2.1.4. Enjoying sport participation

When asked about their participation in *Game Changers*, all students shared that they enjoyed the program, and many asked when it would continue (“yes, we really liked it,” “when can we go back to the gym,” and “can we go back to the gym please”).

#### 4.2.2. Focus groups with champion-participants

One focus group of three champions from Jones Academy participated in the pre- and post-*Game Changers* focus group interviews and one focus group of three champions from Kent Academy participated in the pre- and post-*Game Changers* focus group interviews. The pre-*Game Changers* interviews highlighted two salient themes: perceived barriers to sport participation and limited sport opportunities. The post-*Game Changers* interviews highlighted five salient themes: opportunities for all-student engagement, improving awareness of including students with disabilities in sport, “doable” inclusion, human resource needs, and sustainable game changing programs.

##### 4.2.2.1. Perceived barriers to sport participation

Champion-participants at Jones and Kent Academies recognized several structural barriers related to sport participation. These included limited human resources (“getting the proper number of chaperones,” “we don't really have any extra time this year to put into any of that stuff for those students right now,” and “sometimes don't have the staffing to be able to work with the students with disabilities on from the Learning Centre”), limited financial resources (“the fees for busing and transportation”), and limited material resources (“access to facilities, so, we don't have a field right now or any green space or anything like that that we can go inside with them at the moment”). Some also shared some additional structural barriers related to logistical constraints (“getting the paperwork filled out”), limited teacher/coach awareness/expertise (“we didn”t really understand how [Unified Sports] would all work so there was an issue there where we didn’t really understand how it would all work… so if just kind of fell to the wayside” and “a lot of people don't have experience with people with any kind of disability and that makes people very nervous about the system and getting into the school sport participation”), and safety concerns (“safety and things are the concern for people who are facilitating and in charge because you have such different levels of athleticism and cognitive ability and everything else during their play so I think discomfort with people who in charge”). These structural barriers were virtually identical to many identified by others, including limited accessible information, inadequate facilities, a lack of trained leaders, and prohibitive costs (4, 24, 27–29). (It was noteworthy that the *student-participants* did not recognize these same sorts of barriers; only champion-participants shared/recognized them.)

##### 4.2.2.2. Limited sport opportunities

Given these barriers, the champions also shared that there were limited sport opportunities for their students (“it's almost like there are no options,” “maybe at 12 or 13 out of 800 some to find a sport that those kids can participate in at a high school level,” and “100%, different opportunities [for students with disabilities]”), though they also recognized that this was largely dependent upon students’ disabilities (“so for certain disabilities I think there are similar opportunities [compared to for students without disabilities]”). The champion-participants' recognition of these limited opportunities (in addition to the barriers preventing them) serves to reinforce the observation that this is unfortunately the “normal” state in many Western education jurisdictions [e.g., (1, 2)]. If only all students were afforded full and equal participation in (sport in) education.

##### 4.2.2.3. Opportunities for all-student engagement

Champion-participants at Jones Academy found that the *Game Changers* program enabled them and others to see opportunities to engage with their schools' students with disabilities. Others have found the same and have positioned this potential outcome as one worth seeking and celebrating [e.g., see (14, 15)]. Creating sport programs whereby students with disabilities would engage with the broader school population had observable benefits for all involved. For example, one champion explained,

through *Game Changers* and having my leadership students with the Learning Centre students, I started to realize that they communicate a lot more than I thought they would in a normal situation. So, one example is when we were on the bus coming on the way to sit down for our field day, we had all the Learning Centre students at the front of the bus and then the leadership students were at the back of the bus, and they were kind of two separate groups. And then after the day on the way home, it was the students who were buddied up together. We're all sitting together on the bus, and they were just talking and laughing the whole way home. And it was I was surprised at how much interaction they had between them.

##### 4.2.2.4. Improving awareness of including students with disabilities in sport

Asked to speak to *Game Changers* as a possible “mechanism” for improving upon people's awareness about how to best include students with disabilities, the champions at one site (again, only at Jones Academy) saw an obvious positive impact (“It's changed the awareness and understanding of Learning Centre students. You can see that shift in their minds, like that smaller group of people and I can play and hang with them and do everything the same as everyone else”). This valuable outcome from having students without disabilities participating in inclusive sport (positively impacting them and the community/society more broadly) agrees with the observations shared by Hassan et al. (14). Contrarily, champions at Kent Academy were forthright in sharing that there were fewer opportunities for others within their school community to similarly benefit from such improved awareness—as few-to-no others were involved with the implemented *Game Changers* program.

##### 4.2.2.5. “Doable” inclusion

Champion-participants also recognized that including students with disabilities in school sport was a vision more easily realized in some activities than in others. For example, one champion explained,

and they start to realize, maybe the kids aren't going to be participating as an athlete on the team, but there's options for them to be part of the team and not necessarily compete as on the varsity basketball team. But maybe with track and field, it's great because there's an opportunity for every kid to be able to compete and be part of the team.

And, recognizing this scenario, champions also realized that they could capitalize on different opportunities if they wished to make school sport experiences more accessible to all students. Most specifically, recognizing the opportunities to be found in Unified Sports, one champion offered,

so, there's that difference, but there's different opportunities to have different things. So, I think by trying to make more opportunities available, that that's something that we can add with the Unified Sport[s] and with the inter-school field days is something that that can all be a part of, and they can feel like they're part of a team and just they enjoy having the other kids around.

Adopting this “doable” inclusion view requires a little give and take with respect to one of the earlier observed barriers to providing some genuine sport opportunities to students with disabilities—namely that students with disabilities may only be afforded “exhibition” competition opportunities at sport events (6, 7). So, in some instances (e.g., track and field), current structures enable authentic competition opportunities. But in others (virtually all other activities chosen), sport seems to be exhibition only. So, while many might aim for only those sport activities that are not exhibition ones, we see occasions to aim high while also taking full advantage of more exhibition-like opportunities (and we see them as sometimes more appropriate).

##### 4.2.2.6. Human resource needs

Although champions were clear in explaining that the *Game Changers* program was rewarding (for themselves and their students with disabilities), they were also keenly aware that having available human resources was critical to offering sport programming for students with disabilities. For example, while all offered high praise for the contributions of their schools' EAs (“there's a lot of work the EAs do, a lot of work to keep them involved” and “we got to see all the things EAs do to support students”), champions at one site (Kent Academy) shared that a lack of engagement and leadership from their peers (i.e., teachers and administrators) had a limiting impact on sport experience possibilities for students with disabilities (“there's not the people in the building to take all those additional sports for the children with disabilities like there's no one was offering to help or assist,” “[we] really took on the role… more like the only ones so there's like no one else,” and “we know how little people will actually do for that side of sport [in this school]”). Just as Kang et al. (27), Martin Ginis et al. (24), and Jaarsma et al. (4) found human resource needs as a barrier to physical activity participation for children and youth with *physical* disabilities, these champions have found the same for students with cognitive and/or intellectual disabilities. Or, as Shields and Synnot (25) found, “people make the difference” (p. 5).

##### 4.2.2.7. Sustainable game changing programs

Though there was much to celebrate related to engaging students with disabilities in sport-related programs, there was a worry amongst champions at one site (Kent Academy) that without the *Game Changers* accompanying financial contribution (participating sites received over $5,000), it was likely things would simply return to normal (“without *Game Changers*, things will probably the same as before” and “they’re just isn’t the same opportunities [without *Game Changers*], they’re very limited”). Another champion elaborated,

having *Game Changers* in the school there's no other opportunity. Funding is needed and it's crazy to think that if we even want to try to do this again next year where's all that funding going to come from? There's just nothing for them [students with disabilities]. *Game Changers* kind of opened her [i.e., the school's principal] eyes to that, to be like we have funding to do this so we must do it but if we didn't have the funding how would we ever do it because there's nothing else?

This structural barrier is one of the greatest facing these student-participants and the school communities in which they are situated. Notably, Martin et al.'s (29) systematic review of barriers to physical activity participation for students with disabilities found financial costs *to the individual* to be especially prohibitive. And, while the *Game Changers* grant funding to school communities mitigated this barrier, the funding was a one-time offering and so the school communities will need to face this hurdle, alone, moving forward.

### 4.3. Sport observations

Students at Jones Academy participated in several sport-related activities, including dodgeball, dance, basketball, swimming, track and field, bowling, field day, Unified Sports, biking, and yoga. The number of students at each of these activities ranged from 10 to 13. Researcher-participants observed track and field (100 m, shot put, long jump) and a field day. Students at Kent Academy participated in several sport-related activities, including basketball, volleyball, bowling, yoga, swimming, biking, open sports, and hiking. The number of students at each of these activities ranged from 11 to 14. Researcher-participants observed an open sports day and yoga. In all observed activities, the champions (and EAs) were critical determinants of the program's success. They had built relationships with their students, knew their histories, and enthusiastically engaged with them.

Champion-participants at Jones Academy actively ensured the inclusion of the students. For example, students were provided with school jerseys, they sat with other track and field team members, and were offered snacks and a lunch. They were greeted with “fist bumps” and were cheered on throughout the events, and they clearly enjoying their interactions with the champions. EAs at both sites were highly engaged, actively participating alongside the students. EA participation during open sports, basketball, and yoga encouraged student engagement, and added to student enjoyment. A field day involving Jones Academy exemplified the possibilities for *Game Changers*. Outside on a cold and windy day, students enthusiastically engaged in a multi-activity field day organized and led by leadership students from Jones Academy and another school not part of the *Game Changers* program. Highly active, students smiled and laughed as they participated with students from the other school and the leadership students. Students often initiated play and were reluctant to leave the playing field at lunchtime. Aside from the genuine engagement with the students, organization and planning appeared to be critical to the success of this event and others.

When observed during an open sports day at Kent Academy, some students were left alone, and others needed prompting to engage in an activity of their choice. Students required and enjoyed the engagement of the champions and EAs. At Kent Academy, yoga was not as successful as other activities. Although EAs participated alongside the students, this activity (led by an outsider) was not suitably adapted or modified for student success, resulting in disengaged students unable or unwilling to follow the (hired) instructor.

## 5. Discussion (“Take Away” learnings and game changers moving forward)

Again, the *Game Changers* participatory action research had three idealized goals: (a) to bring to the fore para/adapted/inclusive sport opportunities for all students; (b) to provide an empowering opportunity for students with disabilities to participate, make choices, and act as leaders in the development of sport programming; and (c) to engage youth with disabilities in sport as participants, leaders, mentors, and role models.

### 5.1. Sport opportunities for all students?

Certainly, *Game Changers* did bring to the fore increased sport opportunities for the students. This was true at both sites, though it seemed to be especially true at Kent Academy—where, without *Game Changers*, there were no other sport possibilities for students with disabilities. Notwithstanding this encouraging finding, it is important to consider this “success” alongside *Game Changers*' initial focus upon enabling *sport* participation opportunities in interscholastic sport programs or intramural sport programs. For example, of the 10 “sport” offerings at Jones Academy (dodgeball, dance, basketball, swimming, track and field, bowling, field day, Unified Sports, biking, and yoga), a number might be more appropriately labelled recreational or leisure pursuits (e.g., dance, biking, yoga). Similarly, of the eight “sport” offerings at Kent Academy (basketball, volleyball, bowling, yoga, swimming, biking, open sports, and hiking), some are similarly recreational/leisure pursuits (e.g., yoga, swimming, hiking).

So, the *Game Changers* program did offer students *some* additional opportunities to engage in sport experiences, in both interscholastic sport programs (e.g., track and field) and intramural sport programs (e.g., Unified Sports). This goal was met. That recreational/leisure pursuits were also made available to students is a positive outcome, though not an anticipated one. So, future iterations of *Game Changers* (i.e., the soon-to-occur subsequent cycle in a new school context) might (re)consider this “sport” focus. If increased participation in interscholastic sport programs or intramural sport programs is to remain a main or primary goal, then more focused attention must be placed upon this it. Alternatively, moving forward, *Game Changers* might consider improving upon sport and recreation/leisure pursuit opportunities as complementary worthwhile goals of the program.

While those within school communities with *Game Changers* programming will ultimately have the agency to make these distinctions and decisions themselves, we suggest that a focus upon *sport* participation (rather than sport and/or recreation/leisure pursuits) would be a worthier goal. That is, we are not advocating for movement only, and we had not (by design) considered the barriers and benefits related to recreation/leisure pursuits. Rather, our purposeful focus has been paced upon school sport experiences (again, in both interscholastic sport programs and intramural sport programs). Providing recreation/leisure pursuit options/alternatives does not address the initial issue deserving of action (research). Students with disabilities want, need, and deserve school sport opportunities and offering other movement possibilities instead of bona fide sporting ones “others” these students.

It is also important to note the students' self-reported physical activity increased only on weekends and lunch hours. Certainly, the lunch hour increase was partly due to *Game Changers* programming during some lunch hours. It was odd, though, that students' self-reported physical activity did not increase in school sport, given the purposeful focus of *Game Changers*. These students may not have viewed their *Game Changers* participation as bona fide school sport participation—a reasonable assumption given, for example, the recreational/leisure focus of some pursuits alongside existing school sport programs. Really, only track and field and Unified Sports offered something resembling interscholastic programs (though all other activities could be viewed as intramural sport programs).

### 5.2. Leaders in the development of sport programming

Through their initial participation in the one-day participant workshop (alongside the champions), the students were able to play a role in the co-creation of innovative and responsive solutions to their own challenges related to sport participation. The initial one-day participant workshop, built upon principles HCD, provided authentic opportunities for students to play an active role in facing participation barriers and identifying the sports (and recreation/leisure pursuits) they wished to take part in. With the supportive leadership of the champions (and hands-on engagement of multiple EAs), these students played a role in their own programming. They recognized a (sport participation) gap in their own school community and co-constructed a contextually relevant one for themselves. In fact, it would be fair to say that they played a greater role in this sort of exercise than did any of their peers in their school communities. That is, no other groups of students were afforded the same sort of agency to co-create their own sport-related experiences. This finding also aligns with the survey finding affirming that their autonomy was greater after the *Game Changers* experience.

### 5.3. Participants, leaders, mentors, and role models?

It is with respect to *Game Changers*' third idealized goal (i.e., to engage youth with disabilities in sport as participants, leaders, mentors, and role models) that there is perhaps the greatest space for improvement moving forward. Though the students did engage in sports (and, again, in recreation/leisure pursuits) as participants (and as leaders who co-chose sport experiences), we do not believe they were able to engage in authentic opportunities to act as mentors and/or role models. Ongoing opportunities to act as leaders, apart from the initial workshop day, were also observably absent.

Here, it is important to explain that *Game Changers* was introduced to school communities (as potential partners) as a sport-related program for students with disabilities—with no expectations about the type or nature of the disabilities. For example, *Game Changers* was a possible program for students without Individual Education Plans (IPPs), not enrolled in a Learning Centre class, and without any cognitive or intellectual disabilities. So, a *Game Changers* program for students with physical disabilities (only) would have certainly resulted in entirely different sport experience possibilities and accompanying mentorship and role modelling opportunities. But, still, these students also could have taken on some age- and ability-appropriate mentorship and role modelling experiences related to their sport and recreation/leisure pursuits. For example, they might have taken on some leadership with some younger students from a neighboring elementary school, or with family members, or with a true peer-group. Undoubtedly, some (if not most) participants could have taken on some genuine leadership and role modelling responsibilities, particularly if they were supported by champions and EAs.

On the other hand, on its own, providing meaningful sport and recreation/leisure pursuit experiences was a heavy and important undertaking—particularly at Kent Academy where nothing similar had existed before. So, again, future iterations of *Game Changers* (i.e., the soon-to-occur subsequent cycle in a new school context) might (re)consider this mentorship and role modelling focus. If these are to remain as priority goals, then more focused attention must be placed upon them. Alternatively, *Game Changers* might consider focusing more upon sport and recreation/leisure pursuit participation opportunities, with such groups of students.

### 5.4. Limitation

Given the range of cognitive and intellectual (dis)abilities of student-participants, gathering and determining their “authentic” input was not always an altogether straightforward task. This was true during the co-construction of sport programs, where some (more vocal and/or verbal) students had more input than did others. It was also true during data collection activities. For example, during focus groups sessions, some students spoke plenty while others said very little (or had EAs, with the best of intentions, attempting to speak for them). Also, given that surveys were only completed with the assistance of a teacher or parent/guardian, it is difficult to know whose voices were really being captured. Certainly, it is important to be transparent about these limitations. However, this can be done while also endeavouring to attend to them and remaining comfortable living with them.

Related to this limitation is our reliance upon verbal methods of data collection. Though we also collected data through observations and surveys (completed with champion-participants or parents/guardians), our focus group interviews relied, almost completely, upon verbal methods (and some student-participants were seemingly near non-verbal). So, future *Game Changers* programming and participatory action research might attend to suggestions offered by scholars such as Clish et al. (60) and Seale et al. (61), who suggest the consideration of additional alternative forms of non-verbal methods for data collection when researching with youth with cognitive and/or intellectual disabilities.

### 5.5. Concluding comments

Only through ongoing informal conversations with champions did we come to see two additional “low hanging fruit” possibilities for providing sport participation opportunities for students with disabilities at both Jones and Kent Academies: offering Unified Sports and offering adapted physical education. On many occasions, champions spoke of Unified Sports as a possible pathway to sport participation. Though not yet a part of either school's normal slate of school sport offerings, 35 other schools within the province do have Unified Sports programs. This Special Olympics program [see (62)] offers inclusive school-based sport clubs, whereby students with and without intellectual disabilities participate in sports activities alongside one another. Both schools should work with Special Olympics Nova Scotia to bring Unified Sports into their school communities. Additionally, many of the students at Kent Academy, we realized, were in the gymnasium for their first time ever during their *Game Changers* experience. This was an especially odd realization for us. Because of their disabilities, some of those students were not able to take physical education with a large class and the school did not have smaller adapted physical education classes. Kent Academy should create and offer an adapted physical education class. Such an offering might consider two additional related suggestions. First, Lieberman et al.'s (63) modified physical education model, like Unified Sports, has smaller classes made up of students with and without disabilities. This “unified” physical education model would enable many students to benefit. Second, an adapted/modified/unified physical education program might also prioritize the Sport Education [see (64)] instructional model, whereby students may derive an authentic sport experience (e.g., seasons, affiliation, formal competition, culminating events, record keeping, festivity) within physical education.

Additionally, though this *Game Changers* participatory action research has yielded important and informative findings for these two school communities (as well as for subsequent *Game Changers* sites), connections and applications with other similar contexts are also possible. And, by similar contexts we mean almost every other school community in the nation—with their own unique populations of students with disabilities. Without any need or effort to replicate the *Game Changers* “model” in an entirely identical manner, the ideas and ideals shared herein certainly warrant attention and possibly encourage consideration-for-implementation elsewhere. Just as students with disabilities exist in virtually every school community, so do champions. We see them everywhere. And any of them can “step up” and work with their students to do this sort of essential work (and, we know, many do). Coordinated efforts like this one, whereby students, teachers, and/or university researchers, together, recognize and respond to a need (e.g., for sport participation) can occur elsewhere. That is, others can work together to plan, act, observe, and reflect, all in an effort to “correct” that which needs attention within their school communities. Such a practice can improve upon the lives of students elsewhere, in similar and additional ways. Our hope is that others will consider this *Game Changers* example, with its demonstrated potential to effect positive change, and will take up similar challenges in their own school communities.

## Note

As observed by Le Clair (65) and Kiuppis (66), and amongst others, there are tensions over language use and conventions related to the terms “disabled people” and “people with disabilities.” These tensions exist within the Disability Studies and Inclusive Education literature (66). “People first” language emerged in the 1980s as a response to the then common manner of objectifying and othering those with disabilities (66). This people first convention also aligns with “person-first” conventions adopted by the United Nations (UN) and its sub-organizations World Health Organization (WHO) and United Nations International Children's Emergency Fund (UNICEF). Herein, we have adopted this people first/person-first use and convention, while also being mindful of the insightful and respectful challenges that have been offered as an alternative to this [e.g., see (67)].

## Data Availability

The original contributions presented in the study are included in the article/Supplementary Materials, further inquiries can be directed to drobinso@stfx.ca.
